# The impact of infrared radiation in flight control in the Australian “firebeetle” *Merimna atrata*

**DOI:** 10.1371/journal.pone.0192865

**Published:** 2018-02-12

**Authors:** Marcel Hinz, Adrian Klein, Anke Schmitz, Helmut Schmitz

**Affiliations:** Institute of Zoology, University of Bonn, Meckenheimer Allee 169, Bonn, Germany; Universitat Bielefeld, GERMANY

## Abstract

Infrared (IR) receptors are rare in insects and have only been found in the small group of so-called pyrophilous insects, which approach forest fires. In previous work the morphology of the IR receptors and the physiology of the inherent sensory cells have been investigated. It was shown that receptors are located on the thorax and the abdomen respectively and show an astounding diversity with respect to structure and the presumed transduction mechanism. What is completely missing, however, is any behavioral evidence for the function of the IR receptors in pyrophilous insects. Here we describe the responses of the Australian “firebeetle”, *Merimna atrata* to IR radiation. Beetles in a restrained flight were laterally stimulated with IR radiation of an intensity 20% above a previously determined electrophysiological threshold of the IR organs (40 mW/cm^2^). After exposure, beetles always showed an avoidance response away from the IR source. Reversible ablation experiments showed that the abdominal IR receptors are essential for the observed behavior. Tests with weaker IR radiation (11.4 mW/cm^2^) also induced avoidance reactions in some beetles pointing to a lower threshold. In contrast, beetles were never attracted by the IR source. Our results suggest that the IR receptors in *Merimna atrata* serve as an early warning system preventing an accidental landing on a hot surface. We also tested if another fire specific stimulus, the view of a large smoke plume, influenced the flight. However, due to an unexpected insensitivity of the flying beetles to most visual stimuli results were ambiguous.

## Introduction

Forest fires cause enormous financial losses in many countries of the world. Even worse, lives of humans as well as of untold numbers of animals are often lost. Due to global warming the problem of devastating wildfires most probably will increase in the coming years. Most important, therefore, is the early detection of an emerging fire before its intensity becomes too high. A look into nature shows that certain insects can be regarded as natural airborne fire detection systems. Those so-called pyrophilous insects approach forest fires and can be found more frequently in the burnt area than in the unburnt area prior to the fire [1]. The community of pyrophilous insects is rather small and consists of about 30 species of beetles, 10 species of flies, 8 species of bugs and 2 species of moths [2]. The main reason for the pyrophilous behavior is reproduction because on a freshly burnt area a favorable food supply for the invaders and their offspring exists. The fire creates huge amounts of fire-damaged and dead wood which is a valuable source of food for the larvae of pyrophilous wood-boring beetles [3–6]. Additionally, the fire-killed wood is the substrate for fast-growing post-fire ascomycete fungi; the food source of pyrophilous flat bugs of the genus *Aradus* [2, 7].

In some pyrophilous species the dependence on fire has increased in a way that a population of those species most probably cannot survive without fires [4, 8]. Therefore, this group of highly fire-adapted insects should have special sensory and behavioral adaptations to detect and approach a fire. Once on the freshly burnt area, insects also should be able to detect hot spots from some distance to avoid landing on surfaces with deleterious temperatures. Two genera of jewel beetles (Buprestidae) can be classified as highly pyrophilous: 11 species of the genus *Melanophila* [9] and the Australian firebeetle *Merimna atrata* which is the only species in its genus [10]. Whereas the *Melanophila* species can be found on nearly all continents with the exception of Australia [11], *Merimna* is endemic to Australia [4, 5] (except for a few records from southern Papua New Guinea, R. Holynski, pers. communication). In both genera extensive sensory adaptations to the pyrophilous biology have been described. First of all, electrophysiological recordings have revealed that beetles can smell characteristic smoke components like α-pinene, 2-methoxyphenol, and furfural with outstanding sensitivity [12–14]. Furthermore beetles are equipped with infrared (IR) receptors. However, receptors are very different in the two buprestid genera. *Melanophila* species have a pair of IR pit organs on the metathorax located behind the coxae of the middle legs. Each pit organ houses about 90 dome-shaped IR sensilla with a diameter of 20 μm [15, 16]. In contrast to this, *Merimna* has one to three pairs of abdominal IR organs located ventrolaterally on the second, third and sometimes also on the fourth abdominal sternite [17, 18]. IR organs are more or less round shallow invaginations of the cuticle and are innervated by a small sensory complex consisting of a large multipolar thermoreceptor and two much smaller scolopidia [19]. Furthermore, a large difference in sensitivity of the IR receptors in *Melanophila* species and *Merimna* has been reported. Whereas in pyrophilous *Melanophila* species a relatively high sensitivity of 100 μW/cm^2^ [20] or–in a theoretical study–even the outstanding sensitivity of about 10 nW/cm^2^ has been reported [21], electrophysiological experiments in *Merimna* yielded a much lower sensitivity of only 40 mW/cm^2^ for the IR receptors [22]. This has led to the hypothesis that *Merimna* does not use its IR receptors for the detection of remote forest fires but for the detection of hot spots when flying over a freshly burnt area [4, 22].

Consequently the smell of smoke and IR radiation could be used by the beetles to detect and approach a fire. Behavioral experiments with *Melanophila cuspidata* walking in a two arm olfactometer have revealed that several compounds emitted from heated wood flakes (*Pinus sylvestris*) were attractive to the beetles [13]. Although very interesting, these results do not allow a final statement about the role of smell of burning during the performance of search flights. The tested odors could also be important when beetles crawl on a burnt stem gathering information about the suitability of the charred tree as a breeding substrate. In conclusion, information about the importance of visible (VIS) and IR radiation in flight is totally missing in IR-sensitive pyrophilous insects.

To elucidate the role of electromagnetic radiation in flight control in a pyrophilous insect we have done experiments with the Australian firebeetle *Merimna*. It was intended to examine how visual cues (e.g. the sight of a smoke plume) and IR radiation affect the flight of the beetle. Regarding IR radiation, we focused on testing the above hypothesis.

## Materials and methods

### Animals

In January 2017 adults of *Merimna* (Gory and Laporte 1837) were collected on burnt areas in and around the Perth metropolitan area in Western Australia. Beetles were caught 16–48 hours after forest fires in Eucalyptus forests (Collecting Permit No.: 01-000129-1, Department of Parks and Wildlife, WA), and exported to Germany (State Export Permit No.: OF000214, Department of Parks and Wildlife, WA and Permit No.: PWS2016-AU-002123 of the Australian Government, Department of the Environment, Canberra, ACT). Because *Merimna* is omnivorous and shows a cannibalistic tendency, beetles were kept isolated in transparent plastic boxes and fed with raisins, peanuts and almonds. Water was given ad libitum. Under these conditions most beetles survived for 3–6 months. The behavioral experiments complied with the *Principles of Animal Care* of the National Institute of Health and with the corresponding laws of Germany (Tier-schutzgesetz).

### Experimental setup

Flight experiments were conducted in a small room (3 x 4 m) at air temperatures between 23 and 26°C. A large window was located in the middle of one wall. Before the start of an experiment, beetles were allowed to acclimatize in the experimental room for 1 hour. Holders appropriate to mount the beetles into the experimental setup and to allow the flying beetle any changes in flight direction were adapted from Auerswald and coauthors [23]. Holders consisted of insect pins (length 35 mm, diameter 0.5 mm) inserted through metal tubes (length 15 mm, diameter 0.8 mm). The insect pins fitted exactly into the tubes but could easily be rotated. In the vertically arranged holders the upper knobs of the pins prevented the pins from falling out of the tubes. The lower ends (1 mm) were bent and with the two-component glue Pattex Stabilit Express (Henkel) small discs were modelled around the hooks. Depending of the size of the tested beetle, discs had diameters of 3–5 mm and their lower surfaces were formed slightly concave. Before an experiment, beetles could quickly be fastened with the pronotum to an appropriate disc by a low melting wax / rosin mixture. The metal tubes of the holders were glued to carbon fiber rods (length 150 mm, diameter 3 mm) which could be mounted with the upper end to an adjustable holding clip in the experimental setup. Finally, two white Tipp-Ex dots were painted on the base of the disc on the pronotum and on the apex of the right elytron. Dots served as position markers for the video tracking software (see below).

**Fig 1:**
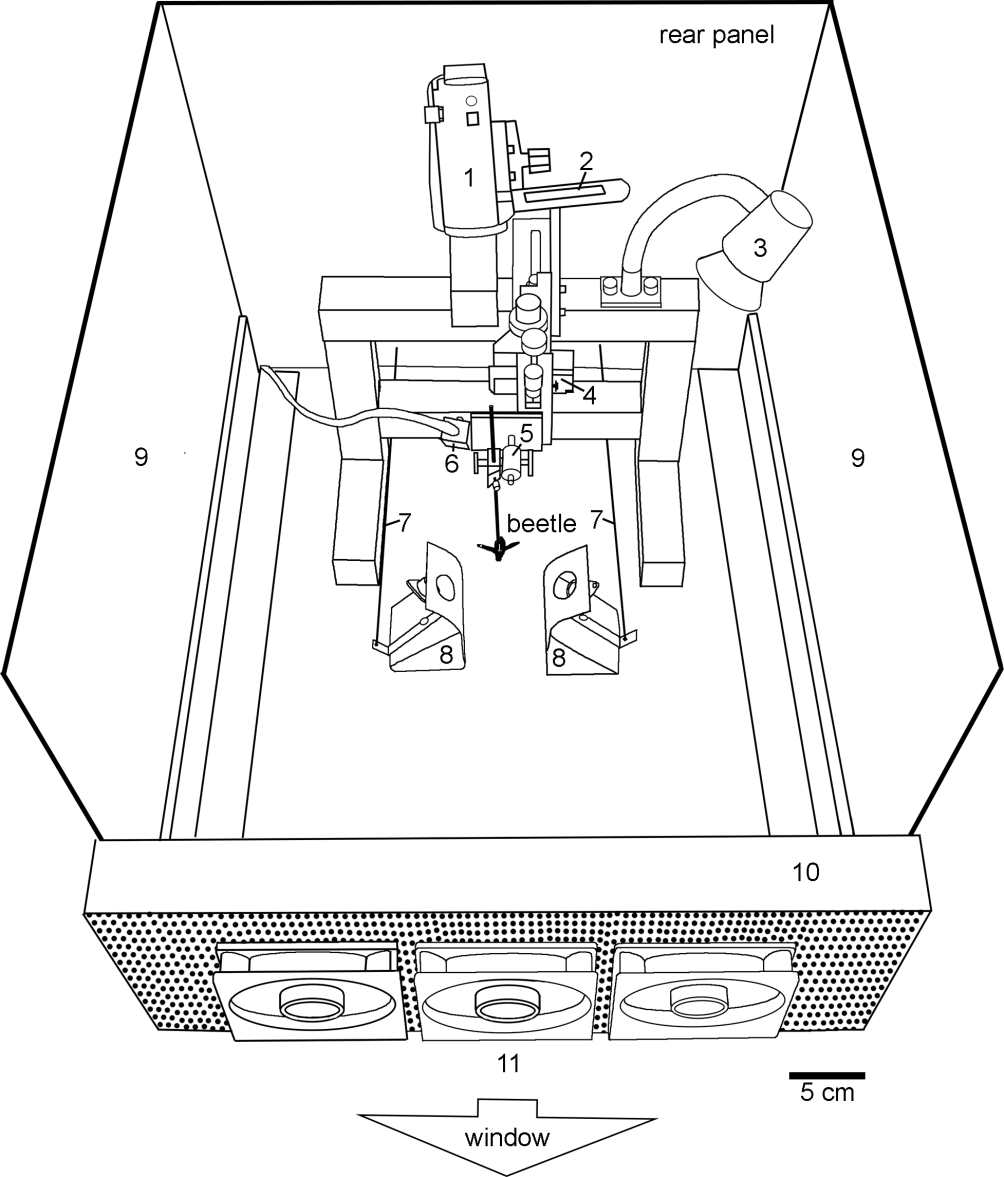
Experimental setup. The setup consisted of the following components:1 video camera, 2 movable screen of the video camera, 3 halogen lamp, 4 triaxial micromanipulator, 5 adjustable clamp for mounting the beetle, 6 box with three LEDs, 7 draw bars, 8 IR emitter mounted behind cover plates, 9 projection screens, 10 laminator, 11 three computer fans. In zero position the beetles flew towards the window against an airflow of 0.8 m/s generated by the fans. The airflow prevented transfer of heated air from the IR emitters to the beetle.

The experimental setup is shown in Fig 1. It was mounted on a table in the middle of the room in front of the window. The experimenter stayed behind the rear panel of the setup and observed the flying beetle directly or on the screen of the video camera (Fig 1, components 1 and 2).

Beetles were fixed in the setup about 13 cm above the ground. In the initial starting position (in the following called zero position) beetles were oriented with the head towards the bright window. Care was taken that beetles could turn left and right without being impaired by any counteracting forces caused by gravity. Thus a perfect symmetrical mounting of the beetle was carried out. For this purpose we adjusted the holding clamp (Fig 1, comp. 5) until the resting beetle, holding a little Styrofoam ball with the tarsi, always maintained its current orientation when the ball had been gently turned around in small steps from -70°to + 70° with respect to the zero position. Afterwards the beetle was aligned to two laterally positioned IR emitters (Fig 1, comp. 8) with a three-axis micromanipulator (Fig 1, comp. 4) until a distance of 70 mm between the aperture of an emitter and the respective side of the lateral abdomen of the beetle was reached. The round steady state black body IR emitters (Model EK-3430, HelioWorks, Inc., USA) had a clear aperture of 14 mm and housed a small coiled Kanthal filament. If supplied with a peak power of 11.84 W the filament glowed red and reached a temperature of 950°C. Emitters had no windows and, therefore, emitted the full unattenuated blackbody spectrum. Emitters were pivot-mounted behind curved cover plates with a central aperture (diameter 24 mm). IR sources could be rotated towards and away from the beetle by the experimenter from outside the arena by drawbars (Fig 1, comp. 7). When an emitter was directed towards the beetle (as shown in Fig 1), IR radiation radiated through the aperture of the cover plate onto the abdomen of the beetles. Radiation impinging on the abdomen of the beetle was measured with a LM-3 HTD thermopile sensor (Coherent Inc., USA) connected to a Fieldmaster FM power meter (Coherent Inc., USA). If emitters were supplied with 11.4 W (default setting used in most experiments) the filament glowed light red and a radiation intensity of 48.7 mW/cm^2^ was measured at the position of the lateral surface of the abdomen. In some experiments we used a lower intensity of 11.4 mW/cm^2^ (no reddening of the filament visible). When the emitter was panned away from the beetle by 90°, no IR radiation could reach the beetle. Furthermore the cover plate blocked IR radiation emitted from the heated side wall of the emitter.

**Fig 2:**
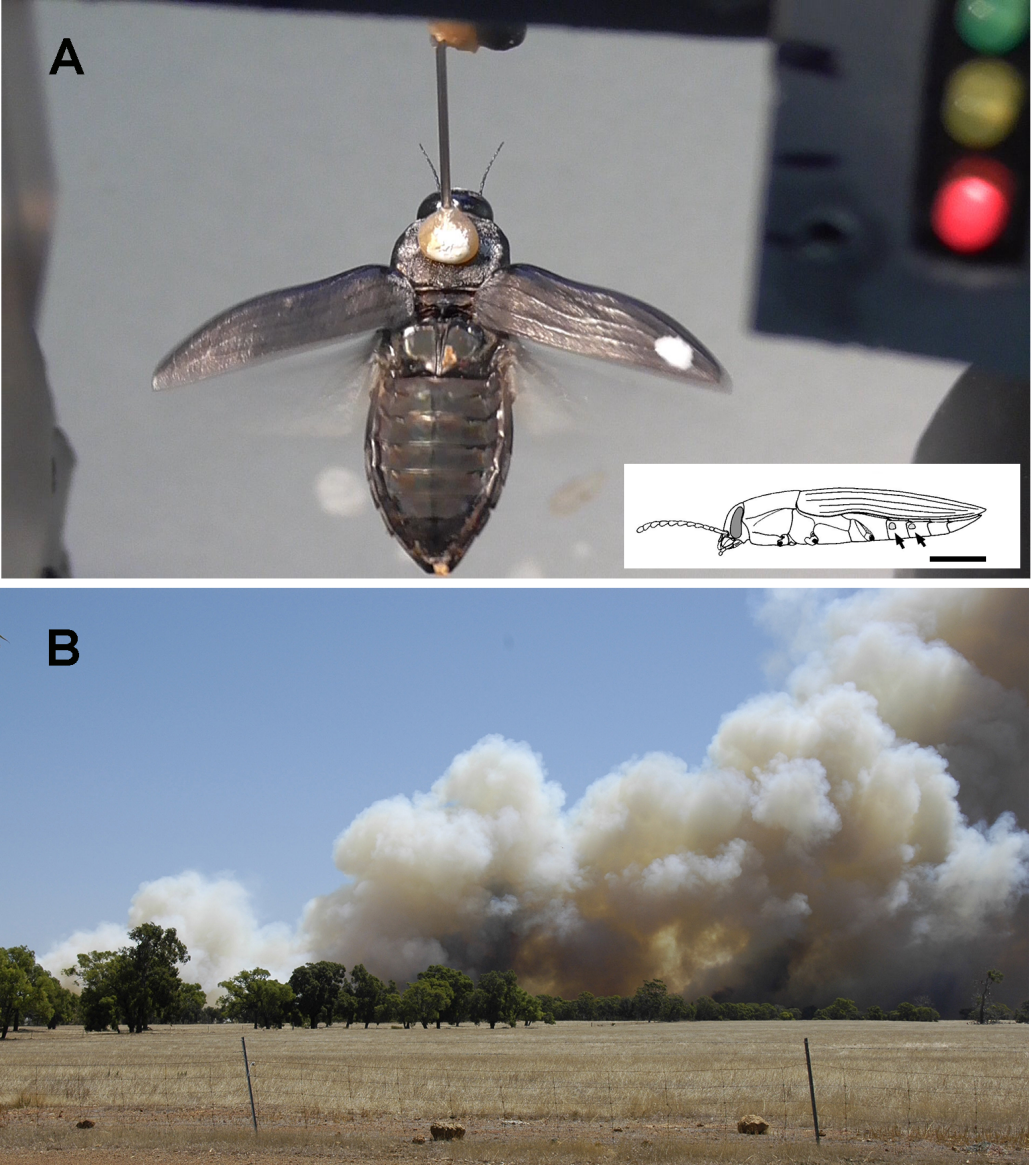
Video recording and presentation of a smoke plume. (**A**) Single video frame showing *Merimna* flying in zero position. White dots (Tipp-Ex) on the pronotum and on the tip of the right elytra served as position markers for the video tracking software. In the upper right corner of the image the LEDs are visible; flashing of an LED indicated the onset of a stimulus. Inset shows position of the IR organs on the abdomen (arrows, bar: 0.5 mm) (**B**) Slide showing a smoke plume above the edge of a forest. The slide could be projected on the projections screens of the setup. The same scenery without a smoke plume could also be projected.

Flying beetles were recorded with a video camera (Panasonic HC-V270, Fig 1, comp. 1) and could be monitored on the inbuilt screen of the camera (Fig 1, comp. 2, Fig 2A) directed towards the experimenter. As shown in Fig 2A, a small array of 3 LEDs was integrated into the upper right corner of the video frames (Fig 1, comp. 6). Onset and termination of a stimulus was marked by pressing a button on a switch box located behind the rear panel of the setup turning a particular LED on or off.

As a highly thermophilic species *Merimna* requires high temperatures [4]. Therefore, a small halogen spot lamp (Halogen Sun Mini, 35 W, Lucky Reptile, Germany, Fig 1, comp. 3) routinely was turned on and directed towards the beetle after the animal had been mounted into the setup. Laterally the setup was bordered by two projection screens (60 x 37 cm) onto which images of edges of the woods with and without a smoke plume of a forest fire (Fig 2B) could be projected. Screens were equipped with rear projection films allowing the view of a projected slide from both sides. Images were projected by two slide projectors installed at a distance of 115 cm outside of the setup left and right from the screens. If not stated otherwise, images of edges of the woods without smoke plume were projected on both screens during all experiments. Beetles flying in the zero position were exposed to a frontal airflow generated by three computer fans (Fig 1, comp. 11). In order to laminarize the airflow the air was blown through a honeycomb structure (Fig 1, comp. 10) built up by closely spaced parallel tubes (diameter 5 mm, length 7 cm). Air speed during the experiments was 0.8 m/s. The continuous airflow also prevented that heated air emanating from the heated housings of the emitters could reach the beetle.

### Experimental procedure

After a beetle had been mounted into the setup and irradiated for some minutes by the halogen lamp it started to walk on the Styrofoam ball. A few minutes later the beetle usually discarded the Styrofoam ball and took off or the ball was gently withdrawn by the experimenter causing the beetle to fly. The halogen lamp was turned off and the video camera on. Now an optomotor stimulus was used to ensure that beetles could easily yaw to the right and to the left. For this purpose a little card (9 x 13 cm) with vertical black and white stripes was semicircularly moved at a distance of about 10 cm around the head and thorax of the beetle. The experiment was only continued if the beetle was able to respond to the stimulus by performing yaw rotations of at least 70° to either side. Sometimes the beetles spontaneously deviated from the zero position. In this case beetles could be easily prompted to return to the zero position by the optomotor stimulus. If the beetle had flown a few dozen seconds in zero position a stimulus was applied (see S1 Video). Onset of a stimulus was marked in the video recording by turning on a predefined LED (green, yellow or red). Stimulus presentation was at least 7 sec. in an experimental trial with an individual beetle a stimulus was repeated at least four times (two times from each side). The following 5 stimuli were presented randomly from left or right:

1. Strong IR radiation (48.7 mW/cm^2^). During the experiments sham experiments were always interspersed; experiments were performed in the same way with the exception that the IR emitter was turned off.
2. Weak IR radiation (11.4 mW/cm^2^)
3. Strong IR radiation (48.7 mW/cm^2^) but aperture of the cover plate blocked by a transparent IR protection filter from a Schott fibre-optic light source (3 x 3 cm sheet of glass, thickness 2 mm).
4. Projection of a smoke plume hanging over the forest on one of the projection screens (see Fig 2B)
5. Introduction of a piece of cardboard (about 30 x 15 cm) shaped like a smoke plume between one of the projectors and the respective screen. The cardboard was held a few centimeters in front of the projection screen with a small rod so that it casted a shadow over the tree tops giving the illusion of a dark smoke plume obscuring the horizon.

As a control, the IR organs of 3 beetles which had responded reliably to strong IR radiation (stimulus 1) were blocked. Then strong IR stimuli were given onto the blocked organs. Blocking was achieved by painting over the lateral region of the abdomen including the IR organs with Tipp-Ex. Before drying a small stripe of customized thin aluminum foil was gently pressed into the drying paint. The foil and the Tipp-Ex could be completely removed after the control experiments to allow further tests with the same beetle. We never observed that beetles were noticeably disturbed by the foil; free moving beetles never tried to remove the foil with the hind legs.

Because of the different kind of stimuli the turning-on of the LED was executed manually by pressing the respective button with one hand. Simultaneously the stimulus was applied with the other hand (e. g. by pulling a draw bar to swing in an IR emitter). This procedure caused inaccuracies regarding the exact onset of the stimulus (approximately in the range of 100–200 ms). However, because reactions of interest lasted much longer (range of several seconds) the results of our study were not influenced negatively by this procedure.

### Data analysis

Inspection and cut of the videos was made with Video Deluxe 2015 Plus software (Magix Software GmbH, Berlin). Videos were cut in sequences with a duration of 10 seconds. Each sequence contained a prestimulus period of 3 seconds followed by a period of 7 seconds starting with flashing of the LED right at the beginning (stimulus onset).

Video sequences were processed with a custom-made video analysis program in Matlab (MathWorks, Natik, MA, USA). The white dots (cf. Fig 2A) were automatically identified in the first frame by the program. A reference vector was computed pointing from the dot on the thorax towards the dot on the tip of the elytron stretched out in a constant position during flight. In each subsequent frame the procedure was repeated. If the vector coincided with the flight direction in zero position, an angle of 0°was allocated. If the flying beetles deviated from the zero position, the resulting change in angle was calculated.

Two different procedures were chosen to evaluate the video files. The first procedure was used in the experiments where strong IR radiation was applied and clear changes in flight direction were observed. First, six video sequences in which the IR stimulus was presented from one side were imported. Then means and standard deviations (SDs) of angles were calculated. The threshold criterion for a change in flight direction after stimulus onset was defined as follows: after application of the stimulus the mean of the angle had to differ from the mean at stimulus onset (3^rd^ second) by the amount of two SDs at stimulus onset. In a few cases the SD showed a pronounced local minimum exactly in the 3^rd^ second. This would have resulted in a very low threshold. To overcome this problem we went back in time a few hundred milliseconds to the next local maximum before stimulus onset. To determine the final deviation from the zero position, the change in angle relative to the zero position was determined 7 s after stimulus onset. This seemed feasible because it has never been observed that a beetle yawed back to the zero position after having changed its flight direction in response to the strong IR stimulus. Furthermore it allowed a comparison of the reactions of all beetles at a defined time.

The second procedure was used in all other experiments where preliminary tests already had revealed that the stimuli obviously were ineffective. Thus, our threshold criterion could not be reached. In the experiments only two or three stimulus applications per body side were given in an experiment and the resulting changes in angles were analyzed for the single flight sequences. Then the mean of the angular changes in the first three prestimulus seconds as well as the mean of the angular changes in the last three seconds (i.e. seconds 7–10) of the video file were calculated. By this procedure the contribution of potential outlier values was minimized. In the next step the angular difference from the two means was calculated revealing a potential reaction to the stimulus in the respective flight sequence. Then a final mean of the angular differences obtained from all trials with a given beetle was calculated; for reasons of clarity the data from left and right stimulus applications were pooled. By this a change in flight direction in response to a stimulus could be quantified and the changes in angle were grouped in six classes. To be able to compare the results of the experiments with the strong IR radiation to the other data, results obtained with strong IR radiation were additionally evaluated according to the second procedure.

### Statistical analysis

For statistical testing the statistics software IBM SPSS Statistics V. 24 (IBM Corporation) was used. First the normal distribution was checked using the Kolmogorow-Smirnov Test. In case of normally distributed data the significance was tested with the t-test. Otherwise the Mann-Whitney U test was used. Data were regarded as significantly different if p was ≤ 0.05.

## Results

### General behavior of the beetles during the experiments

After takeoff in the experimental setup, most beetles remained in a steady flight for several minutes. Flights lasting for 10 minutes or longer (up to 40 min.) were frequently observed. If spontaneous changes in flight direction occurred, changes were never erratic. In fact, beetles deviated from a flight in zero position (cf. Fig 2A) to a certain degree and then continued to fly into the new direction. Usually beetles could be easily stimulated to return to the zero position by moving a small plate with vertical black and white stripes around the head of the beetle (plate also used as optomotor control stimulus, see supplementary S1 Video). If a beetle showed a marked tendency to deviate repeatedly from the zero position, it was excluded from the experiments. However, this was an exception.

A surprising discovery was a marked insensitivity of the flying beetles against most visual stimuli. Although the responses to the optomotor control stimuli were clear, other optical stimuli like movements of the experimenter around the setup or even movements of the experimenters hand in the setup next to the beetle never caused any changes in flight direction. This was convenient for performing the experiments. However, this is in a remarkable contrast to the behavior of the beetles in their natural environment (see Discussion).

### Responses to strong IR radiation

**Fig 3:**
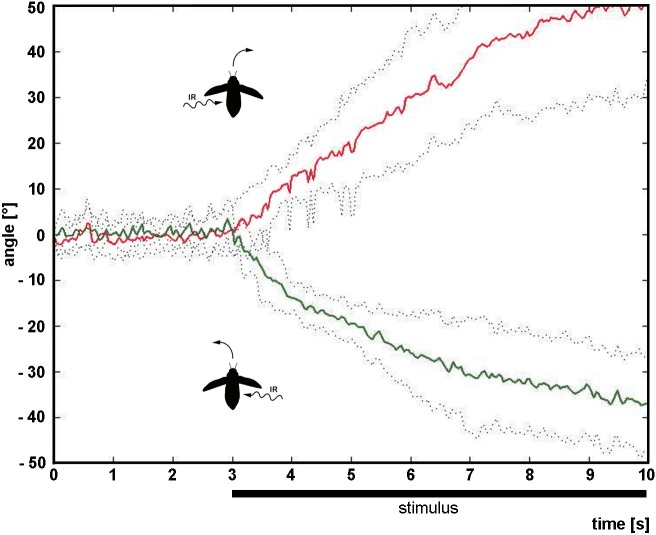
Response of beetle No. 1 to strong IR radiation. Change of direction caused by IR radiation extracted from 10 s video files. After the beetle had flown in zero direction for 3 seconds, the heated IR emitter was swiveled towards the beetle (black bar indicates onset and duration of the stimulus). Mean values (solid line) and standard deviations (stippled lines) of angles of flight direction are shown for 6 exposures to IR radiation from left or right, respectively. If IR radiation was applied from left, beetles turned right (positive angles, red line), if radiation was applied from right, beetle turned left (negative angles, green line). Beetle icons show IR irradiation and the resulting rotation of the beetles.

**Fig 4:**
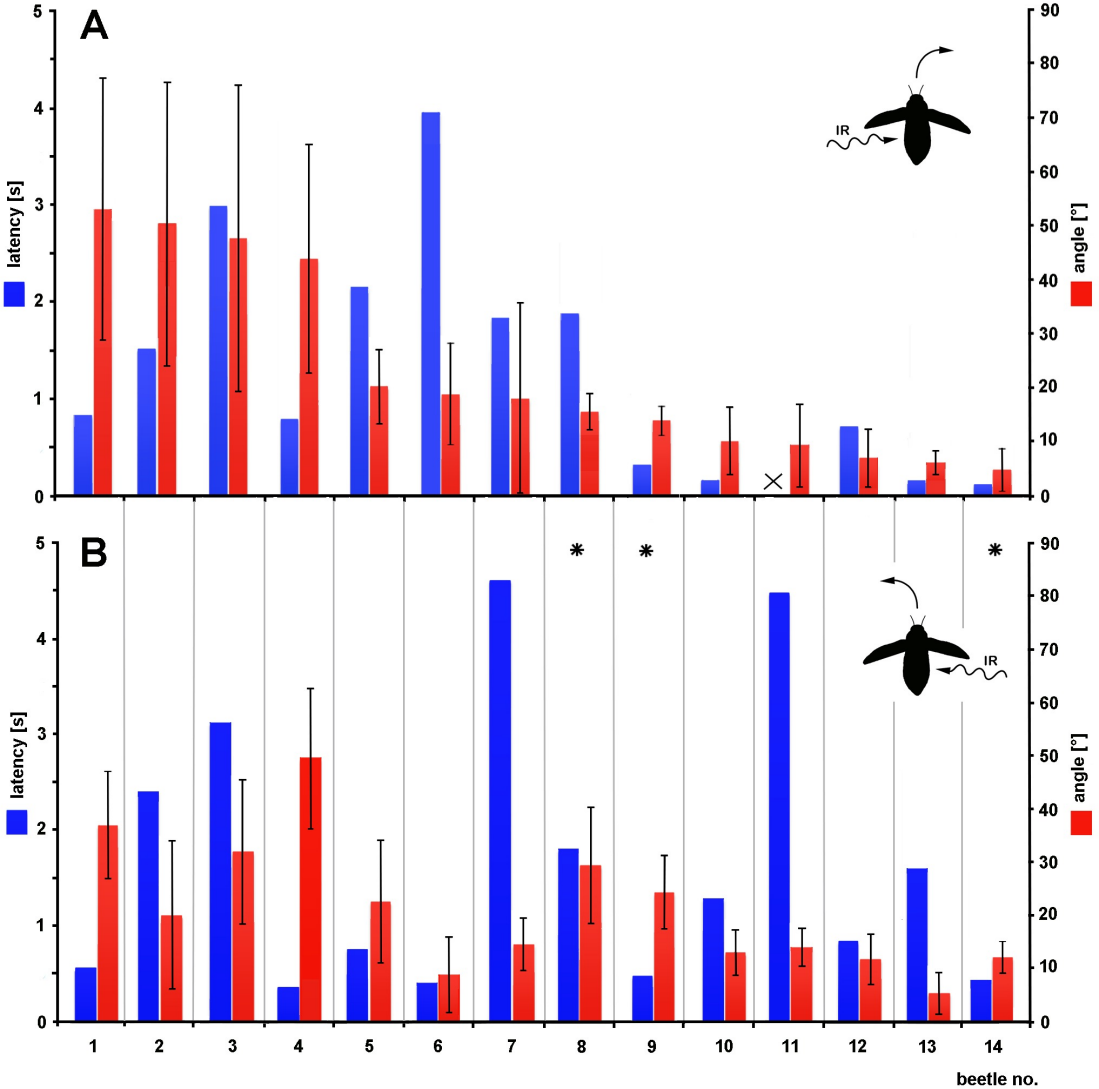
Responses of all tested beetles to strong IR radiation. Responses are given on the left Y-axis (blue bars) as latency until the threshold criterion for a change in flight direction had been reached (mean of the angle had to differ from the mean at stimulus onset by the double SD at stimulus onset). On the right Y-axis (red bars) responses are given as angle of flight direction in the 7^th^ second after stimulus onset. On the X-axis the 14 tested beetles are listed. Significant differences between changes in angle caused by irradiation of the left and right body side exist in beetles 8, 9 and 14 (asterisks, data of beetles 11 and 14 tested with the Mann-Whitney U test, data for all others tested with the t-test, significance level for both tests was p ≤ 0.05; in beetle 14 only 5 stimulus applications per body side were made). In beetle 11 the threshold criterion was not reached when irradiation was carried out from left. (**A**) IR radiation given from the left. (**B**) IR radiation given from the right.

A total of 14 beetles was tested with strong IR radiation (48.7 mW/cm^2^) representing an intensity 20% above the sensitivity threshold of the IR receptors determined in previous electrophysiological experiments [22]. Without exception the beetles responded with evasive maneuvers; it was never observed that a beetle turned towards the IR source. Pronounced responses of a beetle to irradiation from either side are shown in Fig 3 and also in the supplementary S1 Video. Results for all beetles are shown in Fig 4. Although the changes in flight direction were relatively small in some beetles, the threshold criterion was nearly always reached. The only exception was observed in the trials with beetle 11 when IR radiation was given from the left (Fig 4A).

Depending on whether IR irradiation was given from left or right the magnitude of the evasive maneuvers (red bars in Fig 4) was different in most beetles. However, due to the large standard deviations significant differences were observed only in three beetles (No. 8, 9, 14). Furthermore, no obvious correlation between response time (blue bars in Fig 4) and the change in flight direction measured in the 7^th^ second after stimulus onset could be observed (both directions of irradiation considered). Some beetles which needed less than 1 s to reach the threshold criterion showed only small changes in flight direction (e. g. beetles 12 and 14, angles < 20°) but in other beetles much larger changes were observed (e. g. beetles 1 and 4, angles > 35°). On the other hand beetles which need 1.5 s or longer until the criterion was reached showed small changes in flight direction (e.g. beetle 7, angles < 20°) as well as larger changes (beetle 3, angles > 30°). Notably, in some beetles the response time strongly differed depending on the direction of stimulus application (e. g. in beetles 6, 7 and 13).

Sham experiments (IR emitter turned off) performed with all 14 beetles never caused changes in angles differing from the slight spontaneous changes observed in the straight flight sequences without stimulus.

To test if the red color of the glowing filament of the emitter caused evasive maneuvers, the apertures of the cover plates were blocked by a transparent IR protection filter made of glass with a thickness of 2 mm. We tested 6 beetles, which had responded to strong IR radiation (cf. Fig 4) but we never could elicit any change in flight direction by presenting the heated emitter.

### Reversible ablation experiments

**Fig 5:**
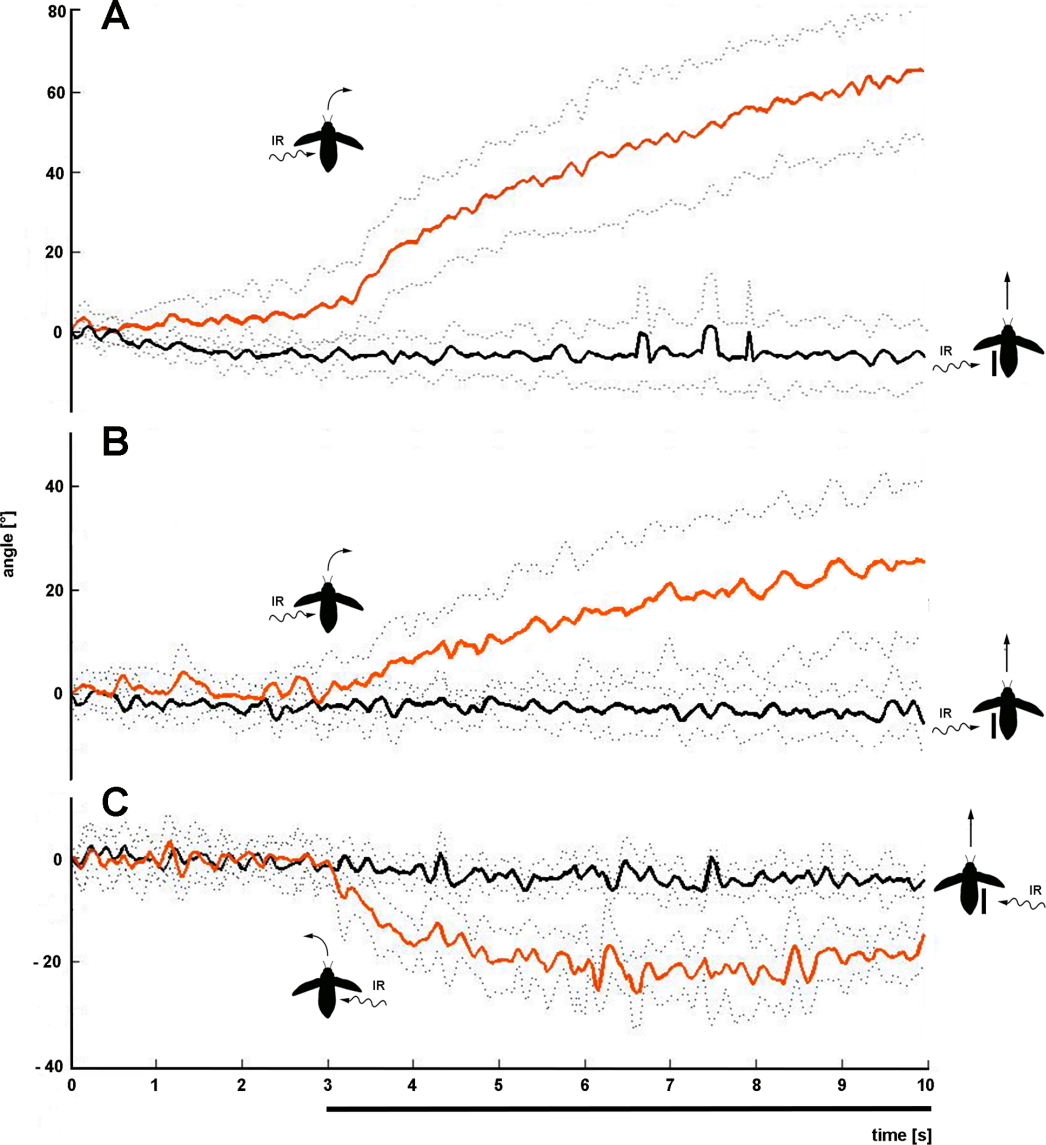
Behavior with blocked and unblocked IR organs. The IR organs of 3 beetles which clearly had responded to strong IR radiation were covered with Tipp-Ex and aluminum foil. No reactions could be discovered (black traces, threshold criterion not reached). After removal of the covering beetles were tested again and responded to IR radiation as before (red traces, threshold criterion reached). n = 6 stimulus applications per body side. In all beetles significant differences exist between the means of the angles (calculated according to the second evaluation process, t-test, p ≤ 0.05). (**A**) Responses of beetle 4. No significant difference in strength of response was measured compared to the earlier results shown in Fig 4 (t-test, p > 0.05). (**B**) Responses of beetle 15 (not included in Fig 4). (**C**) Responses of beetle 8. No significant difference in strength of response was measured compared to the earlier results shown in Fig 4 (t-test, p > 0.05). Bar indicates exposure to IR radiation.

To test the hypothesis that the abdominal IR organs are the sites of IR perception we covered the lateral sides of the abdomen with Tipp-Ex and aluminum foil. After the tests with the blocked IR organs the Tipp-Ex and the foils were removed. Results showed that beetles with blocked IR organs did no longer respond to the strong IR stimulus. However, after removal of the cover beetles responded as before (Fig 5).

### Responses to the presentation of the smoke plume

**Fig 6:**
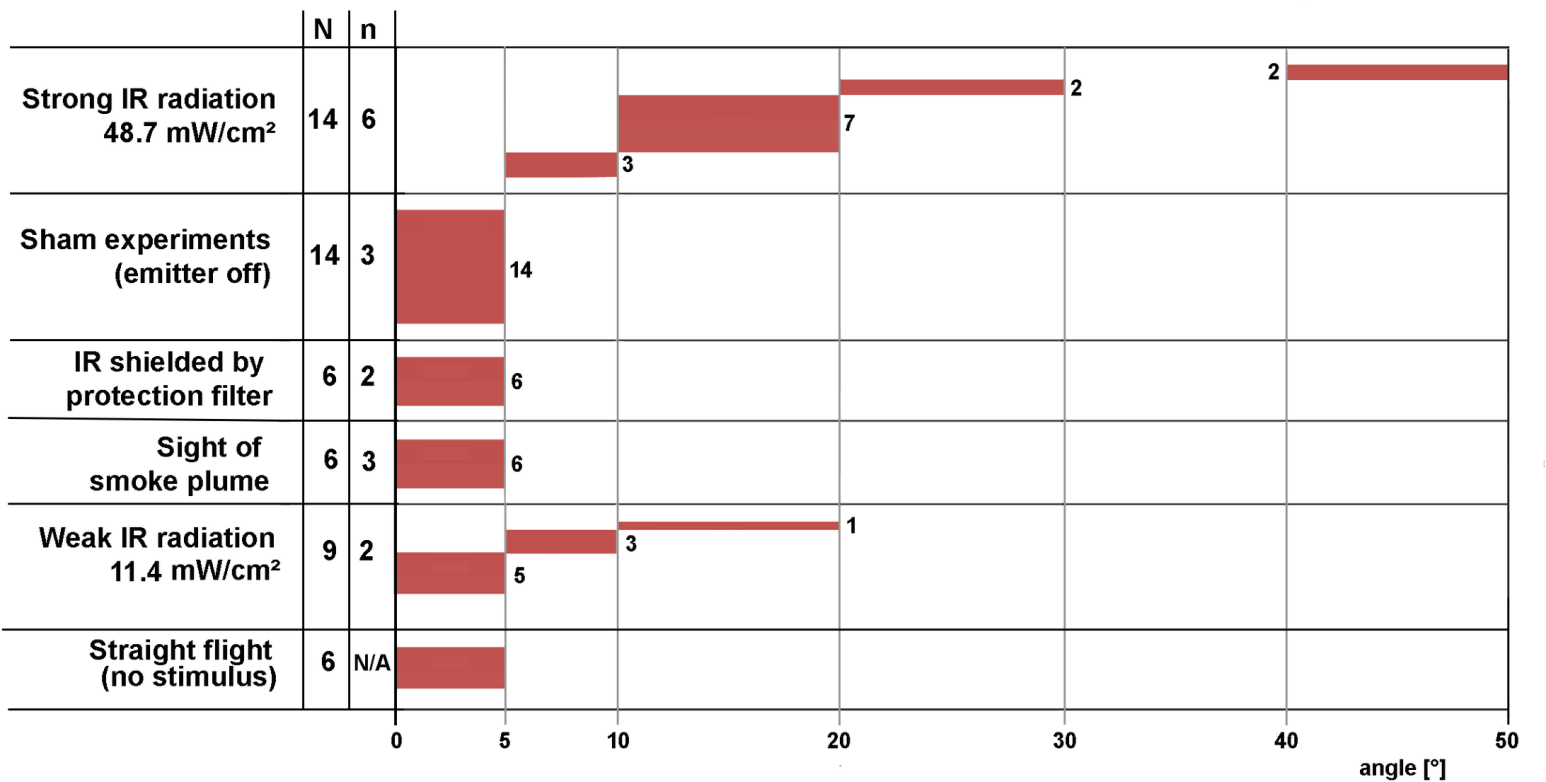
Synopsis of experiments. Results of all experiment are listed excluding the experiments in which the IR organs were covered with aluminum foil (given in Fig 5). Data of the experiments with the 14 beetles tested with strong IR radiation–already provided in Fig 4 - were recalculated to meet the evaluation of the other data (see Material and Methods: last paragraph of Data analysis). Undisturbed flight sequences in the seconds before stimulus presentation were also included (straight flight without stimulus, 4 flight sequences per beetle were evaluated). Changes in flight direction are grouped in 6 classes of angles. N gives the number of tested beetles; n gives the number of stimulus applications per body side.

We never could measure a response of a beetle to the visual presentation of a smoke plume. This was true for the projection of a slide showing a big smoke plume over a forest (cf. Fig 2 B) and also for the casting of a dark cloud–like shadow on a projection screen with a cardboard template (Fig 6).

### Responses to weak IR radiation

The only category of experiments, which revealed ambiguous results were the tests where weak IR radiation was used as a stimulus. We tested 9 beetles and 4 of them showed evasive behavior characterized by changes in angle larger than the changes measured in the other experiments with negative results (Fig 6). As in the experiments with strong IR radiation it was never observed that a beetle turned towards the IR source.

## Discussion

### The abdominal IR receptors trigger thermal avoidance behavior in *Merimna*

So far morphological as well as electrophysiological studies have led to various hypotheses about the possible role of IR receptors in pyrophilous insects. Especially in *Melanophila* beetles, which obviously have sensitive IR receptors, it has been postulated that receptors may serve for the detection of fires from large distances [16, 20, 21, 24]. In contrast, it has been hypothesized that the less sensitive IR receptors of *Merimna* may not be suitable for the detection of remote fires but for the detection of hot spots on a freshly burnt area to prevent a landing on an unfavorable site [22]. According to this hypothesis, the abdominal IR receptors of *Merimna* should serve as early warning systems triggering an avoidance response when the beetle approaches a surface with a deleterious temperature. However, up to now no behavioral data have supported this speculation.

Our results strongly support this function. When we irradiated the beetles with strong IR radiation of 48 mW/cm^2^ the beetles tried to fly away from the IR source without exception. The permanent airflow transverse to the IR emitters excluded that the behavior was caused by convection or conduction of heated air. The intensity of 48 mW/cm^2^ was chosen because it is about 20% higher than the threshold sensitivity determined in previous electrophysiological experiments [22]. An intensity of 48 mW/cm^2^ is about 50% of the total radiation intensity which can be measured when the midday sun is shining from a clear sky. For humans this intensity is perceptible with the skin thermoreceptors; however, it is not painful. Compared to the sensitivity of the IR receptors of IR sensitive snakes (only a few μW/cm^2^ [25]) and *Melanophila* beetles (100 μW/cm^2^ or even only a few nW/cm^2^ [20, 21]) an intensity of 48 mW/cm^2^ is rather high. However, when holding the back of a hand before the heated IR emitter in a distance of 7 cm, the diffuse radiation was hard to feel. Therefore we propose that the observed reaction was not an unspecific pain reaction but a useful thermal avoidance response preventing a potential thermal damage by a hot surface.

A similar thermal avoidance behavior has been measured in a flying non-pyrophilous insect, the migratory locust *Locusta migratoria* [26]. Flying locusts were irradiated from left or right with 250 W heat lamps (Chauffa 40, Globe Pak, Montreal, Quebec). Like in our experiments the distance between the locust and the radiation source was 7 cm and heated air as effective stimulus was also ruled out by interposed IR transmission filters. Unfortunately the intensities of the heat lamps were not given in the publication and we were not able to order a Chauffa lamp from Canada. We, therefore, purchased a similar 250 W heat lamp (Philips IR 250 RH IR2) and measured radiation intensities with our instruments. It turned out that the intensity 7 cm in front of the lamp was 1,140 mW/cm^2^; i. e. more than twenty times higher than in our experiments. In some of their experiments the authors interposed an IR transmission filter to exclude the visible radiation and convection of heated air. However the thermal avoidance reactions were still observed indicating a sensitivity to IR radiation. Again no further information was given about the characteristics of the filter. Because the tungsten filaments in standard heat lamps run at about 2,400 K emitting radiation with a peak output at 1,200 nm [27], most of the radiation is emitted in the infrared. To be on the safe side with an assumption in retrospect, we supposed that 30% of the total radiation was emitted in the visible range of the spectrum (most probably overestimated). Even in this case a radiation intensity of about 800 mW/cm^2^ was irradiated on the locust; still sixteen times higher than in our experiments. To identify a receptor for the observed behavior, the authors made several ablation experiments. It turned out that the compound eyes, the ocelli and also the antennal flagella bearing thermoreceptors [28, 29] were not necessary to trigger the behavior. Finally, the authors discussed the avoidance maneuvers as a possible behavioral artefact and speculated that strong thermal stimulation of wind sensitive hairs on the head of the locust may be responsible for the observed evasive reactions.

Holding a hand 7 cm in front of the 250 W heat lamp caused pain within a few seconds. The locust also had to be exposed for several seconds to the intense IR radiation before a reaction occurred. The authors, therefore, proposed that the cuticular components of a potential thermoreceptor had to be heated up before a sensory cell was excited [26]. The high intensity of the radiation and the relative long latency makes the existence of an IR receptor implausible. We also propose that the observed reaction was a kind of pain reaction not mediated by a special IR receptor.

In contrast, the much weaker IR stimulus applied in our experiments requires a receiver structure with a considerably lower thermal mass allowing quick heating rates. The slightly thinned cuticle in the center of an IR organ [30], the intimate contact of the sensory complex to the cuticle [19, 31] and the system of insulating air sacs below the absorbing area of a receptor [31] all facilitate a fast heating of an abdominal IR receptor in *Merimna*. Thus in our experiment 50% of the beetles responded in less than 1 second to the much weaker stimulus.

Our ablation experiments also support the assumption that the observed evasive reactions are mediated by the abdominal IR receptors located on the second, third and in some beetles also on the fourth sternite [18]. After blocking of the receptors no further responses to IR radiation could be measured. However, after removal of the coverage the responses recovered. Experiments also demonstrate that the observed responses were not mediated by antennal thermoreceptors which *per se* are not suitable as IR receptors [32].

In a recent publication Zermoglio and coauthors reported a similar thermal avoidance behavior in the mosquito *Aedes aegypti*, a major vector of several tropical diseases [33]. Flying mosquitos were also able to determine a deleterious surface temperature of an object (50°C) from some distances and turn away. By their experiments authors were able to demonstrate that reactions were triggered by convection of warm air perceived by sensitive antennal thermoreceptors. Although flying mosquitos showed a similar behavior like *Merimna* when approaching a hot surface, they use a different sensory system. We speculate that a reason may be that mosquitos often approach their hosts in calm surroundings (e. g. warm-blooded hosts resting or sleeping inside a shelter). Here, generation and propagation of warm air gradients is favored and, therefore, perception of heated air by antennal thermoreceptors is an efficient concept allowing host localization even in complete darkness. Simultaneously the mosquito can analyze important host odors. In contrast, on a freshly burnt area after a forest fire very often strong winds occur disrupting any warm air gradients emanating from a hot surface. Additionally, olfaction is not of interest when a beetle just wants to avoid a hot spot. Under these conditions, IR radiation seems to be a more suitable cue because of its total independence of air currents.

Finally the large variations in angular response amplitudes and response times have to be addressed. Large differences were observed between the tested animals (Fig 4). Even in a single beetle both parameters often varied depending on the direction of irradiation from left or right (cf. Fig 4, beetles 2, 6 and 7). Here it has to be taken into account that large changes in flight direction are not necessary when a free-flying beetle wants to turn away from a small non-painful heat source. The beetle will be able to escape after a moderate course correction within few wingbeat cycles. Another possible explanation may be the unknown age of the beetles and the relatively simple construction of the IR organs consisting only of one multipolar thermoreceptor and two scolopidia. In captivity *Merimna* can obtain an age of 6 months. However, because so far *Merimna* cannot be reared we had to catch the beetles in the wild and tested beetles of all ages. We speculate that in older beetles the sensory cells of some IR organs may have lost their sensitivity by degenerative processes or even completely failed. Because in most beetles two IR organs are situated on each body side the overall sensitivity of one side may have been reduced resulting in diminished avoidance reactions.

### Responses to weak IR radiation

In the experiments where IR radiation with an intensity of 11.4 mW/cm^2^ was used, 4 out of 9 beetles showed a moderate but distinct avoidance response (Fig 6). This result was unexpected in two ways because (i) radiation intensity was significantly below the threshold determined in previous electrophysiological experiments and (ii) because initially we expected to attract *Merimna* by a weak IR signal.

The most likely explanation for the observed behavior is that the IR organs have a higher sensitivity than determined by previous electrophysiological experiments. This seems plausible because in the electrophysiological experiments the activity of the multipolar thermoreceptor was recorded by inserting a metal electrode into the cuticle of the IR absorbing area of the IR organ. The metal with its high thermal conductivity most probably sucked heat from the cuticle thereby reducing the sensitivity of the system. Therefore, it seems possible that the sensitivity of the undisturbed IR organ is higher than 40 mW/cm^2^. Consequently, the threshold sensitivity has to be determined by future experiments (see Conclusions below).

Thus our experiments provide some evidence for a higher sensitivity of the IR organs of *Merimna*. However, some beetles still responded with evasive behavior whereas the majority did not change their flight direction. An attractive effect of weak IR radiation could not be shown. In the study of Zermoglio et al mosquitos could be attracted to a heat source that was at host-range temperature (34° C)[33]. Thus a thermal stimulus alone is effective to attract the mosquito to a potential food source. In *Merimna* the situation seems to be different because the thermal stimulus alone may indicate an inappropriate larval food source. The larvae develop only in trees and shrubs belonging to the myrtle family (Myrtaceae) [4, 34, 35]. Therefore, *Merimna* does not approach fires in other types of vegetation (e. g. in *Banksia* forestes) [4]. Because IR radiation does not allow an identification of the burning material, beetles would eventually waste a lot of energy by approaching a fire in the “wrong” forest. This could happen if the beetle approaches a fire downwind or transverse to the wind direction preventing the perception of smell of burning. Therefore, we propose that fire detection in *Merimna* mainly relies on olfactory cues. However, theoretically it can be postulated that the intensity has to be much lower than 11.4 mW/cm^2^ before beetles approach an IR source. This also has to be tested in further experiments.

### The role of visual cues in fire detection

The view of a huge smoke plume emanating from a forest fire is a clear indication for a fire which can be detected from all directions. In contrast to the smell of burning the view of a smoke plume is independent of the wind direction. Therefore, looking for smoke from fire towers or by stand-alone surveillance cameras is currently used by firefighters for early fire detection. For this reason we have proposed that *Merimna* as a diurnal species [4] also might use this easy to perceive cue for fire detection. Numerous observations on freshly burnt areas have shown that *Merimna* has excellent visual capabilities because beetles often flee when the distance to a slowly approaching human is about 15 m or even larger. However, in our experiments the beetles showed a remarkable insensitivity to visual cues. This is clearly in striking contrast to the situation in the field. Although beetles responded to our visual control stimulus–a little card with black and white stripes–by turning towards the card and often also by extending the forelegs in an attempt to land on the card, all other tested visual stimuli were ineffective. The projection of the massive black smoke plume on one of the screens never caused any reaction; beetles always maintained their original flight direction. This was also true for producing sudden high brightness differences on a screen by switching one of the projectors on and off. The reason for the observed insensitivity to those massive visual stimuli remains unclear.

Because of the insensitivity of the tested beetles to most visual stimuli a final decision about the role of a smoke plume in fire detection cannot be made. Currently it cannot be ruled out that the brightness of the projected slide on the projection screens was too low compared to the bright daylight entering through the window or that the two-dimensionality of the scenery prevented the perception of the projected smoke plume as a fire-relevant cue. Furthermore, the tethered mounting of the beetles affected flight performance by reducing the degree of freedom of movement. For instance, a tethered animal cannot control is body angel relative to the flight direction [36]. Additionally, lift production is disrupted [37]. As a consequence, reactions to more subtle signals like stimuli related to reproduction may be suppressed. However, as already discussed above in the context of IR radiation, the sole view of a smoke plume does not provide information about the type of the burning vegetation. This may be a reason that *Merimna* does not use visual cues for fire detection.

### Conclusions

By our results (synopsis provided in Fig 6) we could show that the IR organs in *Merimna* serve as an early warning system protecting the beetles to land on a hot spot. For this reason the position of the IR organs on the ventrolateral abdomen (cf. inset in Fig 2A) seems reasonable because in both sexes the reproductive organs are housed in the abdomen. When the beetles intend to land the ventral abdomen with the IR organs is fully exposed to the potential landing site. Therefore the IR organs can be interpreted as inbuilt non-contact thermosensors of the abdomen protecting eggs and sperm against overheating. Because *Merimna* is active on freshly burnt areas immediately after a fire in the midst of numerous hot spots invisible at daylight such an additional sensory system seems advantageous.

In our experiments we did not distinguish between males and females. The actual reason was that the sex of a beetle cannot be determined by external characteristics. In some beetles we determined the sex after fixation in ethanol by a genital preparation. However, we found no indication for a difference in sensory performance between males and females. As a result, the proposed function of the IR organs is likewise true for both sexes.

Finally the question regarding the sensitivity of the IR organs remains open. Although we imported 50 beetles from Australia we had no information about the age of the beetles. So in the weeks after our return we had to cope with a continuous loss of experimental animals. Because we focused on the experiments described in the *Results* we finally had not enough time for an accurate determination of the threshold sensitivity. This has to be done in future experiments with a focus on sex-specific differences.

## Supporting information

S1 Video Responses of a flying *Merimna* to visual and thermal stimuli. The beetle is attracted by the visual stimulus and tries to avoid the thermal stimulus.
(MOV)
